# Decision Tree Ensemble Method for Analyzing Traffic Accidents of Novice Drivers in Urban Areas

**DOI:** 10.3390/e21040360

**Published:** 2019-04-03

**Authors:** Serafín Moral-García, Javier G. Castellano, Carlos J. Mantas, Alfonso Montella, Joaquín Abellán

**Affiliations:** 1 Department of Computer Science and Artificial Intelligence, University of Granada, 18071 Granada, Spain; 2 Department of Civil, Architectural, and Environmental Engineering, University of Naples Federico II, 80125 Naples, Italy

**Keywords:** data mining, decision tree, novice drivers, road safety, traffic accident, severity, decision rules

## Abstract

Presently, there is a critical need to analyze traffic accidents in order to mitigate their terrible economic and human impact. Most accidents occur in urban areas. Furthermore, driving experience has an important effect on accident analysis, since inexperienced drivers are more likely to suffer fatal injuries. This work studies the injury severity produced by accidents that involve inexperienced drivers in urban areas. The analysis was based on data provided by the Spanish General Traffic Directorate. The information root node variation (IRNV) method (based on decision trees) was used to get a rule set that provides useful information about the most probable causes of fatalities in accidents involving inexperienced drivers in urban areas. This may prove useful knowledge in preventing this kind of accidents and/or mitigating their consequences.

## 1. Introduction

Currently, an estimated 1.27 million people die and 20–50 million people are injured in traffic accidents every year [1], with a devastating human and economic impact. For this reason, it is fundamental to analyze the main causes of the serious severity in traffic accidents to avoid fatal injuries. According to Reference [2], many accidents occur in urban areas. Furthermore, according to Reference [3], the factors that affect road accident severity in urban and non-urban areas are different. A lot of works in the literature are focused on the analysis of this kind of accidents.

As stated by the authors of [4], accidents which occur in intersections have different characteristics, so they must be analyzed separately. Other works [5,6] are focused on accidents that did not occur in intersections, remarking this point.

In addition, many works in the literature, such as References [7,8,9], have revealed that driving experience is an important factor in accident analysis, since inexperienced drivers are more likely to suffer or cause fatal injuries. For instance, References [10] concludes that adolescents, inexperienced in most cases, are not really worried about certain dangers, and that this lack of experience is the reason of many crashes. Further, according to References [11,12], novice drivers have a different perception of risk. Moreover, References [13,14] reveal that novice drivers have less visual attention than experienced ones.

This paper studies accidents in urban areas in Spain involving drivers with 3 or fewer years of driving experience. In particular, our essential goal was to analyze the main causes of fatal injuries in this kind of accidents. This will help road safety analysts and managers to identify the main problems related to inexperienced drivers and take measures to try to reduce the number of accidents of this type and alleviate their consequences. Accidents in intersections were analyzed separately.

The Spanish General Traffic Directorate (DGT) provides three data sets for accident analysis. The first one contains data about the accidents themselves, such as month, weekday, weather conditions, visibility conditions, etc. The second data set contains data about each person involved in the accidents (sex, age, driver yes/no, year the driving license was granted, etc.). The third data set contains data about the vehicles involved in the accidents (type of vehicle, year of registration, and so on).

In the literature, Regression, Logit, and Probit models have been applied for this purpose [15,16,17], providing good results. Nevertheless, these models establish their own dependence relation between variables and their own model assumptions. When these assumptions and relations are not fulfilled, models estimate the severity erroneously [18]. Thus, data mining is commonly applied to traffic accidents databases to extract information and knowledge [15,19,20,21]. Data mining techniques are nonparametric and do not require any previous assumption about the problem.

Often, original data are not ready for knowledge extraction techniques. A preprocessing step is required to get the data in a suitable format for the methods used. This is the most important task in knowledge extraction and the one that requires the most processing time. Specifically, it is necessary to select the relevant features and transform some variables to extract useful information from the data in an easy way. Further, the instances that are not relevant for problem at hand must be filtered out.

In the field of data mining, several techniques have been applied to accident analysis. For example, in References [4,22,23], Bayesian networks were used to analyze the severity of an accident and the dependency relationships between the variables related to the accident. In Reference [24], artificial neural networks were used to research the prediction of the severity of accidents.

Decision trees (DTs) have been applied as well to analyze the severity of accidents [5,6,18,25,26]. DTs provide a very useful model to identify the causes of accidents because they can be easily interpreted and, most importantly, decision rules can be easily extracted from them. These rules can be used by road safety analysts to identify the main causes of accidents.

However, DTs also have some weaknesses. One of their most relevant limitations is that the rules obtained from the tree depend strongly on its structure. More specifically, a DT only allows to extract rules in the sense indicated by the top variables, and so these rules are constrained by the root variable. So, if the root node is changed, the resulting rule set will probably be quite different. Because of this, the information root node variation (IRNV) method was applied to extract suitable rules to identify causes of fatal injuries. This method, which has already been used in References [6,26] for the analysis of severity of accidents, varies the root node to generate different rule sets through DTs. Since the resulting rule sets can be very different, this method returns a final rule set by combining all the rule sets generated by each of the DTs.

Apart from the root node, the main difference between the DTs is the split criterion. In Reference [26], three split criteria were used to build different DTs. One of them is the info-gain ratio (IGR) classic split criterion, which is the criterion used in the C4.5 algorithm [27] and is based on general entropy. The other two criteria are the imprecise info-gain (IIG) criterion [28], based on the imprecise Dirichlet model [29], and the approximate nonparametric predictive inference model (A-NPIM) criterion [30], based on a nonparametric model of imprecise probabilities. In Reference [26], it was shown that using IIG and A-NPIM, a much wider rule set can be obtained than by using only a classic criterion. These two criteria, IIG and A-NPIM, use imprecise probabilities and the maximum entropy measure to select the most informative variables to branching the tree.

The whole data mining process returns a rule set from which the most useful rules must be selected. This selection requires choosing the rules that provide the most general match of the data set (i.e., the rules for which the antecedent is most frequently matched, with corresponding high-frequency matches of their consequent). Two measures are applied in this step: Probability and support.

Therefore, the IRNV method was used with three split criteria (the classical IGR, the imprecise IIG, and A-NPIM) to get a large rule set of accidents in urban areas in Spain. The best rules were selected, and the resulting rule set provides useful information to the Spanish Directorate General of Traffic (DGT) for the identification of the causes of accidents involving novice drivers in urban areas and for raising awareness of these dangers among this group of drivers to help them become more cautious.

Hence, the main aim of this paper was to obtain a rule set about the causes of fatal injuries in accidents of inexperienced drivers in urban areas via the IRNV method with different split criteria. Using this technique with several split criteria, a wide set of rules was obtained. Subsequently, the most useful rules were selected. These rules will help the DGT to take measures to avoid this kind of accidents and/or mitigate their consequences.

The rest of this paper is structured as follows: Section 2 describes the data set, the preprocessing step, and the methods that are used. Section 3 shows the results and includes the pertaining discussion. Finally, Section 4 is devoted to the conclusions.

## 2. Data and Methods

### 2.1. Accident Data

The Spanish Directorate General of Traffic (DGT) collected accident data over a period of 5 years (2011–2015). These data contain three tables per year. One of these tables (accidents) refers to the characteristics of the crashes (weather conditions, time, weekday, and so on). The second table (people) contains data about the people involved in each accident. Finally, the third table (vehicles) covers the information of the vehicles involved in each crash. A description of the meaning of possible variable values for these tables can be found in https://sedeapl.dgt.gob.es.

### 2.2. Preprocessing Step

This subsection describes the preliminary steps required to obtain the final data sets. The data mining algorithms used to extract knowledge was applied to these preprocessed databases.

The first step consists of creating the class variable: The severity of the accident. Following previous studies, such as those in References [5,6], we considered two possible severity states: Accidents with slightly injured people (1) and accidents with killed or seriously injured people (2). A reason we merged serious injuries and deaths is that, in both categories, there are really few instances in comparison with slight injuries. Notwithstanding, in agreement with other works in the literature [4,15,18,31,32], the severity of an accident is defined as the severity of the most injured people in the crash. This variable must be created through the combination of variables that refer to the death toll, the number of seriously injured people, and the number of injured people. It is created as follows: If the number of seriously injured people or the death toll in the accident is greater than 0 (when the crash occurred or within 30 days after the crash), the severity of the accident is labeled as a fatal Injury, i.e., killed or seriously injured. Otherwise, the severity is labeled as a slight Injury.

Since not all the variables will be useful to predict the severity of a crash, it is appropriate to eliminate those variables that we know are not related with the severity. For example, the accident identifier or the year of the accident are unnecessary to predict the fatality.

The aim of this work was to study accidents of inexperienced drivers in urban areas, so the specific area where the accident occurred was not considered. Variables that are not related to urban areas were also removed. Variables totally or partially associated with actions of pedestrians or passengers were not considered either, for obvious reasons. Neither were variables for which most values are missing, or that have two possible values and one of them predominates.

In addition to the variable subset selection step, we grouped some variable values. For instance, instead of distinguishing between the different types of collisions, we only observed that the type of accident was collision. This grouping was required because variables with many possible values often have a negative impact on the performance of data mining algorithms. For the variable corresponding to the section of the week, we distinguished the weekend, which includes Saturday and Sunday, the end and the beginning of weekdays, i.e., Friday and Monday, and the rest of the weekdays: Tuesday, Wednesday, and Thursday. This separation was based on the different patterns of car traffic on weekends and weekdays [33]. The separation of the age variable into these groups was done in consistency with Reference [5]. Further, some continuous variables were discretized. Most of these transformations were extracted from the work of Reference [5]. The most important transformation was the creation of the variable driver_type, which has value 1 if the driver has three years experience or less, and 2 otherwise.

Once the data set with the variables of interest is available, instances belonging to our subject of study are selected. Since we wanted to study the impact of driving inexperience on the fatality of accidents in urban areas, only data corresponding to accidents in urban areas and drivers with three years experience or less were selected.

Accidents in intersections have different characteristics [4], so they are studied separately. To do so, the entries in the resulting data set were divided into those that correspond to accidents in intersections and those that do not.

Table 1 show the variables for both databases, their meaning, and their possible values. For each data set and each feature, the number of instances (N_I) and the number of instances with a missing value (N_Ni) are also illustrated.

### 2.3. Decision Trees

A decision tree (DT) is a predictive model that can be used for both classification and regression tasks. In our case, the class variable has only two states, which means that it is a discrete variable. Hence, in this work, DTs were used to represent classification problems.

DTs are commonly used in the literature because they are hierarchical structures presented graphically. Therefore, they are quite interpretable models. DTs are also simple and transparent.

Within a DT, each node represents a predictor variable and each branch represents one of the states of this variable. Each node is associated to the most informative attribute that has not already been chosen from the path that goes from the root to this node. To select the most informative variable, a specific split criterion (SC) is used. A terminal node (or leaf) represents the expected value of the class variable depending on the information contained in the set used to build the model, i.e., the training data set. This leaf node is created with the majority class for the partition of the data set corresponding to the path from the root node to that leaf node.

**Figure 1:**
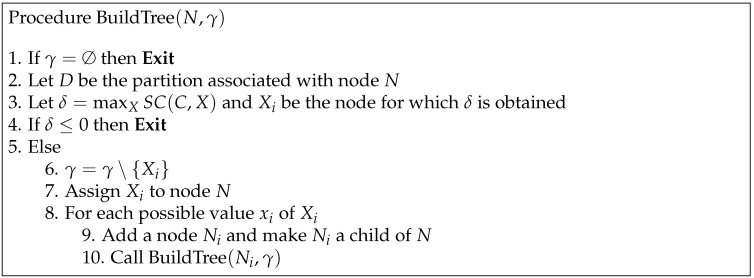
Procedure used for building decision trees (DTs).

DTs are built descending in a recursive way. The process starts in the root node with the full training data set. A feature is selected depending on the split criterion. The data set is then split into several smaller datasets (one for each state of the split variable). This procedure is applied recursively to each subset until the information gain cannot be increased in any of them. In this way, we obtain the terminal nodes. The procedure for building DTs is shown in Figure 1 and described in Reference [28].

To classify a new instance (usually an instance of the test data set) using the DT, the sample values and the tree structure were used to follow the path in the tree from the root node to a leaf.

One important advantage of DTs is that decision rules (DRs) can be extracted easily. A DR is a logical conditional structure written as an “IF A THEN B” statement, where A is the antecedent and B is the consequent of the rule. In our case, the antecedent is the set of values of several attributes, and the consequent is one state of the class variable.

In a DT, each rule starts at the root node, where the conditioned structure (IF) begins. Each variable that appears in the path represents an IF condition of a rule, which ends in leaf nodes with a THEN value, associated with the state resulting from the leaf node. This resulting state is the state of the class variable that has the highest number of cases for the leaf node in question.

### 2.4. Information Root Node Variation

As mentioned above, DTs allow an easy extraction of DRs. However, rules that can be obtained from a single decision tree depend strongly on the variable corresponding to the root node. Thus, within a DT, it is only possible to extract knowledge in the sense indicated by the root variable.

The method proposed in Reference [6], information root node variation (IRNV), varies the root node to generate different DTs. In this way, different rule sets can be obtained, since we have a considerable number of trees with different structure. Therefore, the number of rules in the resulting rule set will be much larger than in a rule set generated using a single DT.

The first step in the IRNV method is to choose a split criterion. Let us suppose that there are *m* features and that $RX_{i}$ is number *i* in the importance ranking according to the split criterion (SC) for i=1,…,m. Then, $RX_{i}$ is used as the root node to build $DT_{i}$ for i=1,…,m. For each of the *m* DTs, once the root node has been fixed, we build the rest of the tree following the same process used for building DTs. After building $DT_{i}$, the corresponding rule set $RS_{i}$ is extracted ∀i=1,…,m. All the rule sets $RS_{i}$ i=1,…,m are added to the final rule set, RS. The process just described is repeated for each SC.

**Figure 2:**
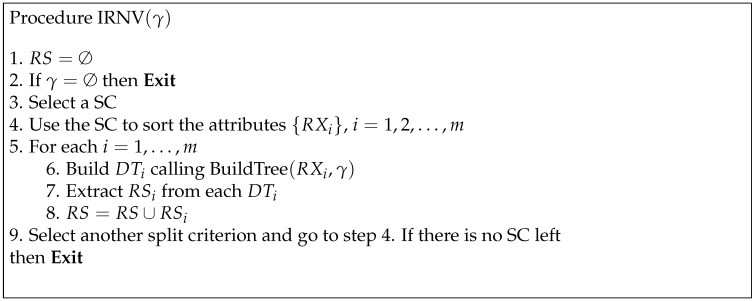
Procedure used to obtain decision rules (DR) using the IRNV method.

A more systematic explanation of the entire procedure is given in Figure 2.

**Figure 3:**
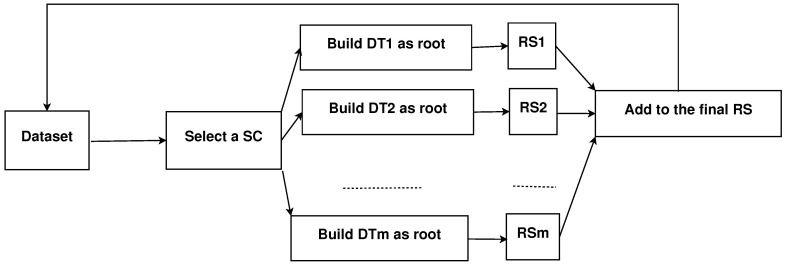
Graphic explanation of the information root node variation (IRNV) method.

Figure 3 illustrates graphically the IRNV procedure for each SC. Keep in mind that the rule sets obtained for all SCs are ultimately combined in a single rule set.

### 2.5. Split Criteria

This section describes the different split criteria used to build the DTs for each root node.

Let D be a data set. Let us suppose that the possible states of the class variable are $c_{1},\ldots ,c_{k}$. Let us consider a variable *X* with values $x_{1},\ldots ,x_{t}$.

The three criteria used are the following.

#### 2.5.1. Info-Gain Ratio (IGR)

The Shannon Entropy [34] for the class is given by Equation (1):

(1) $$H^{D}\left(C\right)=\sum_{i=1}^{k}p\left(c_{i}\right)\log_{2}\left(1/p\left(c_{i}\right)\right),$$

where $p(c_{i})$ is the estimation of the probability of $c_{i}$ based on the data set D (by computing relative frequencies).

In the same way, the entropy for attribute *X* can be defined:

(2) $$H^{D}\left(X\right)=\sum_{i=1}^{t}p\left(x_{i}\right)\log_{2}\left(1/p\left(x_{i}\right)\right).$$

The entropy of class *C* given attribute *X* is given by the following expression:

(3) $$H^{D}\left(C|X\right)=\sum_{i=1}^{t}p\left(x_{i}\right)H^{D_{i}}\left(C|X=x_{i}\right),$$

where $D_{i}$ is the partition associated with value $x_{i}$, i.e., the subset of D in which $X=x_{i}$, and $p(x_{i})$ is the estimation of the probability that $X=x_{i}$ in D, ∀i=1,…,t.

Once the measures given by Equations (1) and (3) have been defined, the info-gain is defined as follows:

(4) $$IG{\left(C,X\right)}^{D}=H^{D}\left(C\right)-H^{D}\left(C|X\right).$$

Finally, the info-gain ratio can be obtained using the IG and the expression given by Equation (2):

(5) $$IGR{\left(C,X\right)}^{D}=\frac{IG{\left(C,X\right)}^{D}}{H^{D}\left(X\right)}.$$

#### 2.5.2. Imprecise Info-Gain (IIG)

The imprecise info-gain (IIG) [28] is based on the Imprecise Dirichlet model [29]. According to this model, for each possible value of class *C*, $c_{i}$, an imprecise probability interval is given instead of a precise estimation:

(6) $$\left[\frac{n_{c_{i}}}{N+s},\frac{n_{c_{i}}+s}{N+s}\right],$$

where $n_{c_{i}}$ is the number of cases in which $C=c_{i}$ in the data set, ∀i=1,…,k, *s* is a given parameter of the model, and *N* is the number of instances in the data set. The higher the value of *s*, the larger the interval will be. Deciding what is the most appropriate value of *s* is not trivial. In Reference [29], the value s=1 is recommended. This work also uses s=1.

This set of probability intervals gives rise to a credal set of probabilities on feature *C*, which is defined in Reference [35] as follows:

(7) $$K^{D}\left(C\right)=\left\{p|p\left(c_{i}\right)\in \left[\frac{n_{c_{i}}}{N+s},\frac{n_{c_{i}}+s}{N+s}\right],\forall i=1,\ldots ,k\right\}.$$

Now, the imprecise entropy $H^{*}$ can be defined as the maximum entropy for the credal set mentioned above. Formally:

(8) $$H^{*}\left(K^{D}\left(C\right)\right)=\max\left\{H^{D}\left(p\right)|p\in K^{D}\left(C\right)\right\}.$$

In a similar way, the imprecise entropy generated by feature *X*, $H^{*}\left(K^{D}\left(C|X\right)\right)$ can be defined as follows:

(9) $$H^{*}(K^{\prime D}\left(C|X\right))=\sum_{i=1}^{t}p\left(x_{i}\right)H^{*}(K^{\prime D}\left(C|X=x_{i}\right))$$

We use Equations (8) and (9) to define the imprecise info-gain (IIG) [28]:

(10) $$IIG{\left(C,X\right)}^{D}=H^{*}\left(K^{D}\left(C\right)\right)-H^{*}\left(K^{D}\left(C|X\right)\right).$$

This paper does not include the computation of the probability in $K^{D}\left(C\right)$ that gives the maximum entropy. A detailed explanation of this procedure can be found in Reference [36].

Unlike info-gain, the value of IIG can be negative. This is an important characteristic since IIG excludes features that worsen the information on the class [37]. In the IRNV method, when a variable has a negative value for the IIG, the rule set obtained for that variable will be empty.

#### 2.5.3. Approximate Nonparametric Predictive Inference Model (A-NMPI)

Let *N* be the number of total instances in the data set, and $n_{c_{i}}$ the number of instances in the data set in which $C=c_{i}$, ∀i=1,…,k. For class *C*, the following credal set of probabilities is considered:

(11) $$K^{\prime D}\left(C\right)=\left\{p|p\left(c_{i}\right)\in \left[\max\left\{0,\frac{n_{c_{i}}-1}{N}\right\},\min\left\{\frac{n_{c_{i}}+1}{N},1\right\}\right],\forall i=1,\ldots ,k\right\}.$$

It should be considered that, in this case, the credal set defined above does not depend on any parameter.

In a similar way to the definition of IIG, the approximate nonparametric predictive inference model (A-NPIM) [30] can be defined as follows:

(12) $$A-NPIM{\left(C,X\right)}^{D}=H^{*}\left(K^{\prime D}\left(C\right)\right)-H^{*}\left(K^{\prime D}\left(C|X\right)\right),$$

where:

(13) $$H^{*}\left(K^{\prime D}\left(C\right)\right)=\max\left\{H^{D}\left(p\right)|p\in K^{\prime D}\left(C\right)\right\},$$

and:

(14) $$H^{*}\left(K^{\prime D}\left(C|X\right)\right)=\sum_{i=1}^{t}p\left(x_{i}\right)H^{*}\left(K^{\prime D}\left(C|X=x_{i}\right)\right).$$

Reference [30] describes the algorithm that returns the probability distribution in $K^{\prime D}\left(C\right)$ with maximum entropy.

### 2.6. Selection of the Best Rules

A priori, a decision rule can be obtained for each leaf node in a DT. However, in a considerable number of cases, some of these rules are not significant because a rule should not necessarily represent a large number of instances of the data set. Rules of this kind do not provide useful information for defining safety measures.

For this reason, sufficiently significant rules are extracted using a mechanism based on two parameters:

- **Support** (*S*): Let us consider a rule of type ‘IF A THEN B’ (A→B). We define support as the fraction of the data set where *A* and *B* are present. In other words, it is the probability that both the antecedent and the consequent occur.
- **Probability** (Pr): It is the probability that the consequent is present given that the antecedent is present. If we have a rule A→B, then $Pr=P\left(B|A\right)=\frac{P(A,B)}{P(A)}$ where P(A,B) is the probability of A∩B.

The selection method consists of choosing only the rules in the set that meet a minimum value (threshold) for both parameters. According to Reference [6], the more suitable threshold values for support and probability depend on some characteristics, such as the nature of the data (balanced or unbalanced), the interest in the minority class, and the data sample. Our two data sets are relatively small (slightly over 30,000 instances) and the nature of our data is clearly unbalanced (less than the 10% of the instances have fatal severity), like the data set considered in Reference [38]. Similarly, we are strongly interested in extracting rules where the consequent is fatal injury. For this reason, we selected the same support and [robability thresholds used in Reference [38]: 10% for *Pr* and 0.1% for *S*. In References [5,6], different thresholds were used: 60% for *Pr* and 0.6% for *S*. However, the data sets used in these works are considerably more balanced.

In other works, such as References [5,6], the rules obtained using the procedure mentioned above were validated using a test set. In this research, validation was not performed due to the imbalance of the data set. If a portion of this data set is extracted for validation, very few rules will be obtained.

## 3. Results and Discussion

### 3.1. Procedure to Obtain the Rule Set

We applied the IRNV method to the two data sets obtained in Section 2.2, respectively, corresponding to intersections and no intersections. More specifically, for each dataset, a DT was applied for each variable and each split criterion. The software used to build the DTs was Weka [39]. We added the necessary methods to build decision trees using the split criteria and the IRNV algorithm explained in previous sections.

Consistently with other works in the literature, such as References [5,6,26], we built the DTs with only four levels to get rules which could be useful, simple and easy to understand for road safety analysts. We did not use pruning to build the DTs. The minimum number of instances per leaf was set to two instances (its default value). Missing values were dealt with by splitting the corresponding instances into pieces (i.e., as in C4.5).

After building the DTs, we extracted the corresponding decision rules to get useful knowledge.

Some of the rules obtained in the previous steps were not valid as they did not match the data. Thus, we only selected the rules that verified the minimum thresholds of support (*S*) (0.1%) and probability (*Pr*) (10%) set in Section 2.6. Since the goal was to determine the causes of fatal injuries, we only selected the rules for which the consequent was fatal injury.

Table 2 and Table 3 show, respectively, the rules obtained for intersections and no intersections following the procedure described above. For each rule, we can see its antecedent. It is given by four columns (*A*_1_, *A*_2_, *A*_3_, and *A*_4_), each one corresponding to one value of some variable (i.e., the antecedent consists of the values of four variables). The consequent (*Co*) is the same for every rule: Fatal injury (*FI*). The last two columns of the tables show the support (*S*) and probability (*Pr*) for the rule. The rules are sorted by probability, so the most interesting rules for analysis are shown first.

### 3.2. Some Remarks about the Rules Related to Accidents in Intersections

As can be seen in rules corresponding to intersections, the kind of accident that produces the most serious severity is running over pedestrians. In fact, in rules 1–9, part of the antecedent is ACT_TY = 2 (the accident type was running over a pedestrian), except for rules 2 and 6 (see Table 1). This shows that inexperienced drivers should pay special attention to pedestrians when they are driving.

Another essential fact is that, in most of the rules, one of the antecedents is SP_IN = 1, i.e., infraction of the driver due to their high speed. This means that driving too fast is a key factor for fatal injuries in accidents involving novel drivers. It can also be seen that antecedent DR_INFR = 5 implies a violation that is not common (see Table 1). It is quite probable that this violation is a consequence of excessive speed. In addition, some rules show that this fact usually produces fatal severity even when the environmental conditions are favorable, such as good weather (rule 3), dry and clean surface (rule 4), good lighting (rule 9) or good visibility (rule 11). Therefore, raising awareness about the importance of speed moderation among people who are starting to drive is critical.

According to rule 1, which implies the highest probability, and rule 5, among others, the situation is more dangerous if there are no pavements. Inexperienced drivers between 21 and 27 years old should be very careful in these cases (rule 14). Drivers over 28 years old should also be cautious in roads with no pavements (rule 21). In addition, the lack of pavements with good environmental conditions can cause accidents with fatal injury (good lighting, rule 16, good state of the vehicle, rule 17, good weather, rule 18, good visibility, rule 20). Hence, novice drivers must be even more careful in roads without pavements. Furthermore, this suggests that building pavements in urban areas will reduce the danger. Further, rule 19 shows that drivers with three years experience or less should be careful between 18:00 and 24:00 while driving in roads without pavements.

There is also evidence that riding motorbikes at night is dangerous for inexperienced riders. In fact, rule 2 shows that motorbike accidents between 0:00 and 6:00 at high speed have a very high probability of fatal injury accident (*Pr* = 68.8%).

Rules 12 and 13 show that it is very important to observe the signals (part of the antecedent in both is DR_INFR = 1). Moreover, excessive speed is part of the antecedent in both rules. Hence, speed moderation is very important. Among other things, driving with moderate speed allows to observe the signals. More specifically, men should pay special attention when the intersection type is “in X o +” (rule 13).

As mentioned previously, the type of accident that causes more fatalities is running over pedestrians. Nevertheless, there are also some rules where part of the antecedent is ACT_TY = 1, (the accident was a collision), although these rules have a lower probability (from rule 23). This means that collisions can also cause fatal injuries. In all these rules, the probability is equal to 1 and the driver made a mistake by not observing a signal (DR_INFR = 1). It means that the driver failed to observe the directions of a traffic officer (see Table 1).

### 3.3. Some Remarks about the Rules Related to Accidents That Do Not Occur in Intersections

In this case, too, collisions with pedestrians are the most frequent type of accident causing fatal injuries. In fact, we see that in most rules, part of the antecedent is ACT_TY = 2 (collision with a pedestrian). Thus, novice drivers should always pay special attention to pedestrians, not only in intersections.

As was the case for the rules corresponding to intersections, most of these rules include SP_IN = 1 (infraction due to excessive speed) and DR_INFR = 5 (a driver infraction which is not common). This means that, in the case of inexperienced drivers, excessive speed usually gives rise to some traffic violation that is not common and often causes a fatal injury. Therefore, it is critical to raise awareness among drivers about the importance of avoiding excessive speed, especially during the first few years.

It can be deducted from some of the rules that the previous points can be aggravated if they are combined with certain circumstances. These circumstances can be driving on Fridays (rule 2) or between 18:00 and 24:00 h (rule 3). Rule 4 shows that excessive speed often triggers accidents of fatal severity in straight road conditions. Furthermore, according to rule 8, men are more likely to suffer fatal injuries because of excessive speed.

Some rules also remark that driving too fast usually produces fatal injuries even with favorable environmental conditions, such as a dry and clean surface (rule 6), good lighting (rule 11) or good visibility (rule 13).

In rules 15 and 17, part of the antecedent is PAV = 0, which implies absence of pavements. It means that the excess of speed in some cases can be aggravated if there are no pavements. Thus, drivers with little experience should be especially careful when there are no pavements.

### 3.4. Summary of the Rules Obtained

Collisions with pedestrians are the type of accident of inexperienced drivers that causes the most fatal injuries in urban areas. Hence, road safety analysts should urge inexperienced drivers to exercise extreme caution to prevent pedestrian accidents while driving.

We have also observed that in most of the accidents with fatal severity involving inexperienced drivers, the speed of the vehicle was excessive. Therefore, speed moderation is crucial for novice drivers, especially during the first few years. Since this is a very common infraction, advertising campaigns should be run to raise awareness about this issue among inexperienced drivers.

The issues mentioned above often give rise to fatal severity in accidents, even when external conditions are good. However, the situation could be aggravated under certain circumstances. These circumstances can be the hour, the weekday and, most importantly, the lack of pavements. This factor can lead to fatal injuries, especially in intersections. Thus, apart from warning novice drivers to moderate the speed in areas without pavements, building more pavements in urban areas (especially in intersections) is desirable.

Another point to consider is that novice drivers must obey traffic officer directions in intersections. They should be aware that the directions given by a traffic officer have priority over the rest of the signals and general rules. Therefore, advertising campaigns should be run to raise awareness about this point among inexperienced drivers.

## 4. Conclusions

This research was focused on the analysis of accidents in urban areas involving drivers with three years experience or less. For this purpose, we used data about accidents provided by the Spanish Directorate General of Traffic (DGT). As a first step, the data were preprocessed for the problem targeted. Next, they were classified into accidents in intersections and accidents that did not occur in intersections. Then, the information root node variation (IRNV) method was applied to obtain a final rule set. These rules were validated to select only those that matched the data and had a fatal injury consequent.

Thus, we created two rule sets for the main causes of fatal injuries in accidents involving inexperienced drivers in urban areas: One corresponding to accidents in intersections and the other one for accidents that did not occur in intersections. From these rule sets, it follows that collisions with pedestrians are the accident type of inexperienced drivers that causes most fatalities. In addition, excessive speed is the main cause of serious injuries in novice drivers. Other circumstances can aggravate the situation; The most relevant one is the lack of pavements. Another important conclusion is that failing to observe the directions of a traffic officer is the cause of some of the fatal injuries in intersections.

Therefore, it is crucial to make inexperienced drivers understand that they should moderate their speed and pay attention to pedestrians, and exercise caution in certain circumstances (e.g., lack of pavements). Increasing the number of pavements in urban areas is also desirable. Finally, inexperienced drivers should be aware of the importance of observing the directions of traffic officers in intersections.

To summarize, in this work, a rule set about causes of fatal injuries in accidents in urban areas with inexperienced drivers was obtained via the IRNV method and a posterior selection process. These rules will be useful to DGT to inform novice drivers about the importance of paying attention to pedestrians and moderating their speed, especially in certain situations, such as where there is a lack of pavements.

## Figures and Tables

**Table 1:** Description of the variables from the resulting data sets.

Feature	Value	Meaning	N_I	N_Ni
SE: Season	1	Winter (between Decenber and February)	7108	6446
	2	Spring (between March and May)	7378	6599
The accident occurred in	3	Summer (between June and August)	7318	6594
	4	Autumn (between September and November)	8204	7692
F_T: Fringe time	1	0:01 and 6:00	2489	2433
The accident happened between	2	6:01 and 12:00	75,112	6780
	3	12:01 and 18:00	11,510	10,406
	4	18:01 and 0:00	8897	7712
S_W: Section Week	1	On Monday	4302	3962
The accident occurred	2	On Friday	4939	4745
	3	Between Tuesday and Thursday	13,598	12,473
	4	On Saturday or Sunday	7169	6151
I_L: Degree of involvement	1	One vehicle	3510	6733
Vehicles involved in the accident	2	More than one vehicle	26,498	20,598
TR_N_INT: Traced no intersection		Missing value		5039
The no intersection tracing of the accident is a	1	Straight line		20,470
	2	Curve		1822
I_T: Intersection type	1	T o X	7028	
	2	X o +	17,882	
The intersection of the accident was	3	A roundabout	4587	
	4	An exit or entrance link	511	
PR: Priority		Missing value	18,134	
	1	Traffic officer directions	18	
	2	A semaphore	3286	
	3	A stop signal	2838	
There was priority indicated by	4	A pedestrian crossing signal	3596	
	5	Road signs	1218	
	6	General rule	591	
	7	Other	327	
RO_SU: Road Surface		Missing value	2032	55
	1	Dry and clean	25,326	23,982
	2	Wet	58	98
The surface was	3	Snowy/Frozen	2364	2863
	4	Oily	34	71
	5	Other state	194	262
LUM: Luminosity	1	In broad daylight	19,701	18,278
	2	In twilight	1280	1377
The accident happened	3	During the night with sufficient road illumination	8965	7483
	4	During the night with insufficient road illumination	62	193
WE_CO: Weather conditions		Missing value	2460	430
	1	Good weather conditions	25,489	24,370
The accident occurred with	2	Fog	61	96
	3	Light rain	1727	2122
	4	Adverse weather conditions	271	313
RES_VIS: Restricted Visibility		Missing value	22,512	20,003
	1	No restrictions	6588	6588
	2	Buildings	29	54
	3	Land configuration	57	77
Visibility restrictions during the accidents due to	4	Weather conditions	60	69
	5	Glare	43	52
	6	Dust	37	30
	7	Other restrictions	416	458
PAV: Pavements		Missing value	262	575
Pavements at the accident scene	0	Yes	28,982	25,400
	1	No	764	1356
ACT_TY: Accident type	1	A collision	25,114	19,621
	2	Running over a pedestrian	1984	3225
The accident was	3	An overturn	500	615
	4	A road exit	1500	2004
	5	Another kind of accident	910	1866
AG_FR: Age group	1	20 years old or younger	7373	6609
	2	21–27 years old	10,673	9695
The driver was	3	28–59 years old	11,401	10,528
	4	60+ years old	561	499
SEX		Missing value	37	26
The driver was	1	A man	22,306	20,338
	2	A woman	7935	6967
MAN: Maneuvers		Missing value	1342	1354
	1	Following the road	7511	
	2	Overtaking	530	726
	3	Turning	2410	761
	4	Entering from another road or access	714	249
The driver was	5	Crossing an intersection	2217	653
	6	Driving in reverse	85	247
	7	Doing an abrupt gear shift	379	880
	8	Doing another maneuver	14,820	13,687
SP_IN: Speed Infraction		Missing value	8801	7694
	1	Too fast	920	1325
The driver was driving	2	Too slow	19	24
	3	At an appropriate speed	20,268	18,288
DR_INFR: Driving Infraction		Missing value	3219	4660
	0	Did not commit any traffic violation	14,014	12,817
	1	Failed to observe a traffic sign	3328	1028
	2	Driving on the wrong side of the road, or invading it partially	165	267
The driver	3	Was overtaking in a forbidden zone of the road	152	188
	4	Failed to observe the security distance	454	1166
	5	Committed other type of infraction	8676	7205
OL_VEH: Old vehicle		Missing value	13,932	9248
	1	Two or less years old	2661	2968
The vehicle was	2	Three or more years old	13,415	15,115
VEH_TY: Vehicle type		Missing value	52	19
	1	A motorbike or equivalent	10,900	9109
The vehicle was	2	A car or equivalent	18,516	17,556
	3	A heavy vehicle	416	518
	4	Another type of vehicle	123	129
AN: Anomaly		Missing value	5484	7602
	1	No	24,281	19,478
Was there any malfunction in the vehicle?	2	Yes	243	251
O_L: Number of passengers	1	Only the driver	20,610	17,329
How many people were in the vehicle	2	Two	4346	3807
	3	More than two	5052	6195
SEV: Severity	1	Slight injury	27,889	25,164
The severity of the accident was	2	Fatal injury	2109	2167

**Table 2:** Final set of rules related to accidents in intersections. Column “NR” is the number of the rule. For all the rules *Co* = *FI*.

NR	*A*_1_	*A*_2_	*A*_3_	*A*_4_	*S*	*Pr*
2	F_T = 1	SP_IN = 1	VEH_TY = 1	DR_INFR = 5	0.1033%	68.8%
3	WE_CO = 1	ACT_TY = 2	DR_INFR = 5	SP_IN = 1	0.23%	59.5%
4	RO_SU = 1	ACT_TY = 2	DR_INFR = 5	SP_IN = 1	0.2166%	58.03%
5	MAN = 1	PAV = 0	SP_IN = 1	ACT_TY = 2	0.1466%	57.89%
6	RO_SU = 1	ACT_TY = 5	DR_INFR = 5	MAN = 1	0.1166%	57.38%
7	DR_INFR = 5	SP_IN = 1	ACT_TY = 2		0.2432%	57.03%
8	I_L = 1	DR_INFR = 5	SP_IN = 1	ACT_TY = 2	0.2266%	56.66%
9	LUM = 1	ACT_TY = 2	DR_INFR = 5	SP_IN = 1	0.1733%	56.52%
10	I_T = 2	DR_INFR = 1	MAN = 8	O_L = 1	0.1033%	56.36%
11	SP_IN = 1	DR_INFR = 5	ACT_TY = 2	RES_VIS = 1	0.2%	55.55%
12	MAN = 8	SP_IN = 1	DR_INFR = 1		0.166%	50.5%
13	ACT_TY = 2	DR_INFR = 1	SP_IN = 1	SEX = 1	0.17%	46.79%
14	AG_FR = 2	PAV = 0	SP_IN = 1	ACT_TY = 2	0.16%	46.6%
15	VEH_TY = 1	ACT_TY = 2	MAN = 1	SP_IN = 1	0.2%	44.03%
16	LUM = 1	PAV = 0	SP_IN = 1	ACT_TY = 2	0.33%	38.17%
17	AN = 1	PAV = 0	SP_IN = 1	ACT_TY = 2	0.45%	37.6%
18	WE_CO = 1	PAV = 0	SP_IN = 1	ACT_TY = 2	0.423%	37.02%
19	F_T = 3	PAV = 0	ACT_TY = 2	SP_IN = 1	0.1766%	36.8%
20	RES_VIS = 1	PAV = 0	ACT_TY = 2	SP_IN = 1	0.397%	34.9%
21	AG_FR = 3	PAV = 0	SP_IN = 1	ACT_TY = 2	0.1866%	32.75%
22	AG_FR = 2	ACT_TY = 2	SP_IN = 1	I_T = 2	0.11%	31.73%
23	SEX = 1	DR_INFR = 1	ACT_TY = 1	PR = 1	0.213%	17.2%
24	RO_SU = 1	ACT_TY = 1	DR_INFR = 1	PR = 1	0.29%	16.86%
25	PR = 1	DR_INFR = 1	LUM = 1		0.24%	16.78%
26	WE_CO = 1	ACT_TY = 1	DR_INFR = 1	PR = 1	0.29%	16.6%
27	AN = 1	ACT_TY = 1	DR_INFR = 1	PR = 1	0.296%	16.45%
28	ACT_TY = 1	DR_INFR = 1	PR = 1		0.296%	16.42%
29	PAV = 0	DR_INFR = 1	ACT_TY = 1	PR = 1	0.296%	16.42%
30	S_W = 3	ACT_TY = 1	DR_INFR = 1	PR = 1	0.11%	14.86%
31	RES_VIS = 1	ACT_TY = 1	DR_INFR = 1	PR = 1	0.246%	14.77%
32	LUM = 1	ACT_TY = 1	DR_INFR = 1	PR = 1	0.183%	14.66%

**Table 3:** Final set of rules related to accidents in no intersections. Column “NR” is the number of the rule. For all the rules *Co* = *FI*.

NR	*A*_1_	*A*_2_	*A*_3_	*A*_4_	*S*	*Pr*
1	SP_IN = 1	ACT_TY = 2	DR_INFR = 5	MAN = 8	0.117%	71.11%
2	S_W = 3	ACT_TY = 2	DR_INFR = 5	SP_IN = 1	0.314%	64.18%
3	F_T = 4	ACT_TY = 2	DR_INFR = 5	SP_IN = 1	0.245%	62.03%
4	TR_N_INT = 1	ACT_TY = 2	DR_INFR = 5	SP_IN = 1	0.574%	59.25%
5	LUM = 3	ACT_TY = 2	DR_INFR = 5	SP_IN = 1	0.179%	59.03%
6	ACT_TY = 2	DR_INFR = 5	SP_IN = 1	RO_SU = 1	0.56%	58.85%
7	MAN = 1	ACT_TY = 2	DR_INFR = 5	SP_IN = 1	0.38%	58.76%
8	SEX = 1	ACT_TY = 2	DR_INFR = 5	SP_IN = 1	0.46%	58.6%
9	AN = 1	ACT_TY = 2	DR_INFR = 5	SP_IN = 1	0.59%	58.06%
10	DR_INFR = 5	ACT_TY = 2	SP_IN = 1	O_L = 1	0.49%	58%
11	LUM = 1	ACT_TY = 2	DR_INFR = 5	SP_IN = 1	0.377%	57.54%
12	I_L = 1	DR_INFR = 5	ACT_TY = 2	SP_IN = 1	0.56%	57.52%
13	ACT_TY = 2	DR_INFR = 5	SP_IN = 1	RES_VIS = 1	0.5%	56.61%
14	VEH_TY = 2	ACT_TY = 2	DR_INFR = 5	SP_IN = 1	0.465%	55.95%
15	ACT_TY = 2	SP_IN = 1	DR_INFR = 1	PAV = 0	0.135%	54.41%
16	VEH_TY = 1	SP_IN = 1	DR_INFR = 5	F_T = 1	0.113%	53.45%
17	S_W = 1	ACT_TY = 2	SP_IN = 1	PAV = 0	0.143%	43.82%
